# Synergistic Inhibitory Effects of Cetuximab and Cisplatin on Human Colon Cancer Cell Growth *via* Inhibition of the ERK-Dependent EGF Receptor Signaling Pathway

**DOI:** 10.1155/2015/397563

**Published:** 2015-09-28

**Authors:** Dong Ju Son, Ji Eun Hong, Jung Ok Ban, Ju Ho Park, Hye Lim Lee, Sun Mi Gu, Jae Yeon Hwang, Myung Hee Jung, Dong Won Lee, Sang-Bae Han, Jin Tae Hong

**Affiliations:** ^1^College of Pharmacy and Medical Research Center, Chungbuk National University, Cheongju, Chungbuk 361-763, Republic of Korea; ^2^Korea Health Industry Development Institute (KHIDI), Cheongju, Chungbuk 363-951, Republic of Korea; ^3^Ministry of Food and Drug Safety (MFDS), Cheongju, Chungbuk 361-709, Republic of Korea

## Abstract

The purpose of this study was to evaluate the anticancer efficacy of cetuximab combined with cisplatin (combination treatment) on colon cancer growth, as well as its underlying action mechanism. Combination treatment synergistically potentiated the effect of cetuximab on cell growth inhibition and apoptosis induction in HCT116 and SW480 cells. Combination treatment further suppressed the expression of the activated form of epidermal growth factor receptor (EGFR) and MAP kinase (p-ERK and p-p38) and also significantly inhibited the activity of activator protein-1 (AP-1) and nuclear factor kappa B (NF-*κ*B). Additionally, the expression of cyclooxygenase-2 (COX-2) and interleukin-8 (IL-8) mRNA was significantly reduced by the combination treatment as compared to the expression seen for treatment with cetuximab or cisplatin alone. We found that the synergistic inhibitory effects of cetuximab and cisplatin on AP-1 and NF-*κ*B activation, as well as on cell viability, were reversed by pretreatment with an ERK inhibitor. Results demonstrate that combined treatment with cetuximab and cisplatin exerts synergistic anticancer effects on colon cancer cells and also suggest that the ERK pathway plays a critical role in these effects *via* the suppression of the EGFR signaling pathway, along with the inhibition of COX-2, IL-8, and AP-1 and NF-*κ*B.

## 1. Introduction

Colon cancer is one of the most common forms of cancer worldwide and is the third leading cause of cancer death in the United States [1, 2]. Its treatment requires a multimodality approach, which comprises surgical resection of the tumor followed by chemotherapy and/or radiation therapy. Despite substantial progress being made in the therapy of colorectal cancer, there is still a need for improved treatments and novel concepts that include the targeted regulation of cancer signaling pathways [3].

The epidermal growth factor receptor (EGFR) has been shown to be overexpressed in several solid tumors [4–6], especially in colon cancer (about 80% of patients) [7, 8], and has also been shown to mediate resistance to chemotherapeutic agents [9]. In addition, the blockage of EGFR results in a significant growth inhibition of several cancer cell lines derived from human carcinomas [10]. Therefore, EGFR is likely to affect multiple aspects of tumor growth and chemoresistance [11]. Since the time EGFR was identified as a cancer target, a number of clinically approved monoclonal antibodies (mAbs) have been developed [12–16]. One of the most promising current strategies involves the use of an EGFR mAb, either alone or in combination with conventional cytotoxic modalities, such as chemotherapy or radiotherapy [17]. As is common with other clinically relevant mAbs, EGFR-targeting mAbs have shown limited effects as monotherapies and are therefore usually administered in combination with radiotherapy or chemotherapeutic drugs [12, 13, 18, 19]. Therefore, the combination of EGFR mAbs with chemotherapy could be effective for the treatment of colon cancer.

Cetuximab, an anti-EGFR immunoglobulin G1 chimeric mAb, has been approved and widely used in the clinical treatment of colorectal carcinoma, as well as cancers of the head and neck [20–22]. Cetuximab combined with radiotherapy significantly improved overall patient survival at 5 years, compared with radiotherapy alone, in head and neck cancer [23]. The survival benefits associated with the addition of cetuximab to first-line chemotherapy for advanced non-small-cell lung cancer expressing high levels of EGFR were also reported [24]. In addition, some preclinical studies suggest that cetuximab inhibits the proliferation of colon cancer cell lines expressing EGFR and enhances the antitumor activity of chemotherapy and radiotherapy [25]. While these treatment combination options have improved the survival of patients, additional nontoxic targeted treatment options are needed in the therapy-refractory setting of advanced colon cancer.

*cis*-Diamminedichloroplatinum(II) (cisplatin) is one of the most potent antitumor agents known and displays a broad spectrum of antitumor activities, including the treatment of colorectal cancer [26]. Its cytotoxicity is mediated by its interaction with DNA (deoxyribonucleic acid) to form DNA adducts, which activate several signal transduction pathways leading to the activation of apoptosis [26, 27]. However, it also has severe adverse effects, including nephrotoxicity, peripheral neuropathy, and ototoxicity [26, 28, 29]. Therefore, there is a need for the continuous development of new drugs and improved therapeutic approaches for colon cancer treatment. Current research has mainly focused on combinations of chemotherapy drugs to reduce or eliminate the negative side effects of chemotherapies to treat colon cancer [30]. For example, combination therapy with 5-fluorouracil and cisplatin was shown to be more effective and less cytotoxic than therapy with 5-fluorouracil alone in human colon cancer cells [31]. Interestingly, the combination of an EGFR inhibitor with cisplatin showed synergistic inhibition effects on cisplatin-resistant chondrosarcoma cells [32]. However, the potency of cetuximab combined with cisplatin in colon cancer had not been previously studied. Thus, in the present study, the anticancer efficacy of cetuximab combined with cisplatin on colon cancer cell growth and its action mechanisms were investigated.

## 2. Materials and Methods

### 2.1. Cell Culture

The human colon cancer cell lines HCT116 and SW480 were obtained from the American Type Culture Collection (Manassas, VA, USA). They were cultured in Dulbecco's modified Eagle's medium (DMEM, Gibco-BRL) and Roswell Park Memorial Institute medium 1640 (RPMI-1640, Gibco-BRL), respectively, with 10% heat-inactivated fetal bovine serum (FBS) and penicillin/streptomycin (100 U/mL) at 37°C in a humidified atmosphere containing 5% CO_2_ in a CO_2_ incubator. Cells were plated in 100 mm culture dishes at 4 × 10^5^ cells for the subsequent experiments.

### 2.2. Cell Growth Measurement by MTT Assay

Cell growth was assessed by a colorimetric metabolic activity assay using 3-(4,5-dimethylthiazol-2-yl)-2,5-diphenyltetrazolium bromide (MTT) solution. In brief, cells were seeded in 96-well plates at 1 × 10^4^ cells/well and cultured for 24 h. Cells were then treated with or without cetuximab (30 to 100 *μ*g/mL) or cisplatin (1 to 5 *μ*g/mL). Following incubation for 24 h, the drug-containing medium was removed and replaced by 100 *μ*L of fresh medium, and then 20 *μ*L of 0.5 mg/mL MTT solution was added to each well. After incubation for 1.5 h, the medium with MTT was removed and 200 *μ*L of dimethyl sulfoxide (DMSO) was added to each well. The plates were then gently agitated until the color reaction was uniform, and the colorimetric evaluation was performed with a microplate reader at 540 nm.

### 2.3. Apoptosis Analysis by TUNEL Assay

Apoptotic cell death was determined by observing morphological changes and with the terminal deoxynucleotidyl transferase-mediated dUTP nick end labeling (TUNEL) assay as previously described [33]. Briefly, cells were cultured on a glass chamber slide (BD Biosciences, Franklin Lakes, NJ, USA) and cultured for 24 h, and then cells were treated with cetuximab or cisplatin alone or in combination. After incubation for 24 h, cells were washed with phosphate-buffered saline (PBS), fixed with 4% paraformaldehyde in PBS, and processed for TUNEL staining by using in situ Cell Death Detection Kit (Roche Diagnostics GmbH, Mannheim, Germany) according to manufactures' instructions. Cells were counterstained using 4′,6-diamidino-2-phenylindole (DAPI) and mounted using fluorescence mounting medium. Samples were imaged using a fluorescence microscope (200x magnification). The total number of cells (DAPI-positive cells) in a given area was manually counted, and apoptotic cell death was calculated as the percentage of TUNEL-positive cells out of the total number of cells.

### 2.4. Western Blot Analysis

Whole cell lysates, cytosolic extract, and nuclear extract were obtained as previously described [33]. Sodium dodecyl sulfate polyacrylamide gel electrophoresis (SDS-PAGE) and Western blot analysis were performed as described previously [33, 34]. Briefly, cells were cultured in 6-well culture plates at 5 × 10^5^ cells/well and cultured for 24 h. Cells were then treated with cetuximab or cisplatin alone or in combination for 24 h. Cells were washed twice with PBS and were lysed, and the proteins were separated on 10% to 15% SDS-PAGE. The proteins were transferred to polyvinylidene fluoride (PVDF) membrane, and membranes were blocked with 5% skim milk in TBS/T-buffer (Tris-buffered saline with tween 20) for 2.5 h at room temperature. The protein-transfer membranes were proved with the following primary antibodies: mouse monoclonal antibodies directed against EGFR, phosphorylated p38 mitogen-activated protein kinases (p-p38 MAPK), extracellular signal-regulated kinase (ERK), p65, p-I*κ*B, *β*-actin, and histone-H1 (1 : 1000 dilution); rabbit polyclonal antibodies directed against p-EGFR, p-ERK, p50, I*κ*B, c-Jun, c-Fos, cleaved caspase-3, and cyclooxygenase-2 (COX-2) (1 : 1000 dilutions). Protein expression was visualized by a chemiluminescence reagent (Amersham Pharmacia Biotech, Inc., Buckinghamshire, UK) and detected using a digital chemiluminescence imaging system equipped with a charge coupled device (CCD) camera (Fusion-FX, Fisher Biotec, Ltd., Wembley, Australia).

### 2.5. Total RNA Extraction and RT-PCR

Total RNA was extracted using the RNeasy Mini Kit (QIAGEN GmbH, Hilden, Germany) according to the manufacturer's instructions. We first performed the reverse transcription polymerase chain reaction (RT-PCR) experiment to synthesize complementary DNA (cDNA) using a WizScript RT Master (Wizbiosolutions Co., Seongnam, Korea) according to manufacturer's instructions. PCR was then performed with cDNAs of interleukin-8 (IL-8) and *β*-actin, primers, and Taq DNA polymerase. The primers used were as follows: IL-8 sense primer (catalog number: N-1065, Bioneer Co., Daejeon, Korea) and *β*-actin sense primer (catalog number: N-1080, Bioneer Co.); the amplicon size was 300 bp for IL-8 and *β*-actin. Reactions were carried out in an automatic thermal cycler (Eppendorf Instrumente GmbH, Hamburg, Germany) using the following protocol: 10 min at 95°C, then 35 cycles of denaturation for 30 s at 95°C, annealing for 30 s at 60°C, and extension for 20 s at 72°C. PCR products were electrophoresed on a 1% agarose gel in TAE buffer (Tris-acetate-EDTA buffer) and visualized by ethidium bromide staining.

### 2.6. DNA-Binding Activity Assay by EMSA

DNA-binding activity of the activator protein-1 (AP-1) and nuclear factor kappa B (NF-*κ*B) was determined using an electrophoretic mobility shift assay (EMSA) as described previously [33]. In brief, cells were cultured in a 100 mm dish at 37°C for 24 h and then treated with cetuximab or cisplatin alone or in combination. After incubation for 24 h, cells were washed three times with ice-cold PBS and their nuclear extracts were prepared for EMSA. The relative density of the DNA-protein binding bands was scanned using densitometry and quantified by the Lab Works 4.0 software (UVP Inc., Upland, CA, USA).

### 2.7. Calculation of Combination Index (CI)

The combination index (CI) value was analyzed using the CompuSyn 1.0 software (ComboSyn Inc., Paramus, NJ, USA) as described previously [35]. A CI value less than, equal to, and more than 1 indicates synergy, additivity, and antagonism, respectively. The CI equation is also the basis of the fraction affected (fa) versus CI plot in the CompuSyn software. The utility of the fa versus CI plot lies in that it covers all effect levels, 1%–99% inhibition, for a given combination. Therefore, to determine the level of synergy and/or antagonism of the two-drug combination, fa versus CI plots were generated for HCT116 and SW480 cell lines at the same concentrations used in the cell growth measurement.

### 2.8. Statistical Analysis

Statistical analyses were carried out using the GraphPad Prism 5.0 software (GraphPad Software Inc., La Jolla, CA, USA). Pairwise comparisons were performed using one-way ANOVA Dunnett's tests. Data are presented as mean ± standard deviation (SD) of the indicated number of experiments. A *P* value less than 0.05 was considered statistically significant.

## 3. Results

### 3.1. Effects of the Combination Treatment of Cetuximab and Cisplatin on Human Colon Cancer Cell Growth

**Figure 1:**
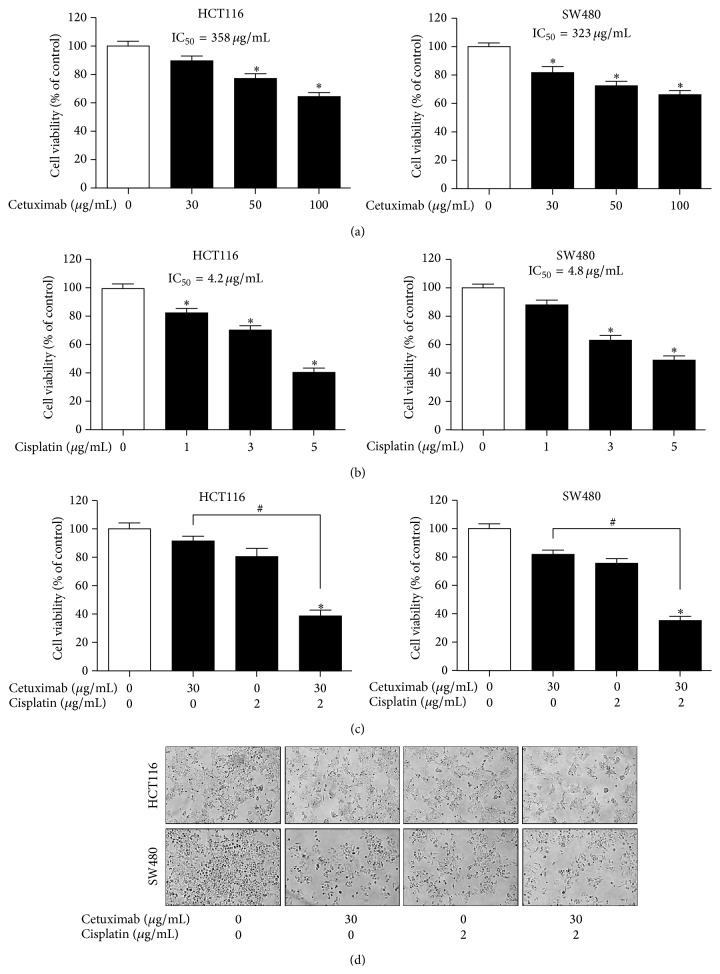
Effects of cetuximab and cisplatin on human colon cancer cell growth. After treatment of (a) cetuximab (30, 50, and 100 *μ*g/mL) or (b) cisplatin (1, 3, and 5 *μ*g/mL) for 24 h, its effect on cell growth was determined by MTT assay in HCT116 and SW480 human colon cancer cells. (c) Cells were treated with cetuximab (30 *μ*g/mL) or cisplatin (2 *μ*g/mL) or the combination of both agents. After treatment for 24 h, cell growth was determined by MTT assay. Data are shown as the mean ± SD of three independent experiments. ^*∗*^ *P* < 0.05 indicates statistically significant differences from the control. ^#^ *P* < 0.05 indicates statistically significant differences from the cetuximab treatment alone. (d) Morphologic observation. Representative images of each experimental group are shown.

The inhibitory effects of cetuximab and cisplatin on cell growth were tested in HCT116 and SW480 cells. Treatment of cetuximab alone for 24 h inhibited cell growth of HCT116 and SW480 cells in a concentration-dependent manner, with IC_50_ values of 358.0 and 323.4 *μ*g/mL, respectively (Figure 1(a)). Treatment of cisplatin alone for 24 h also inhibited cell growth in a concentration-dependent manner, with IC_50_ values of 4.2 and 4.8 *μ*g/mL in HCT116 and SW480 cells, respectively (Figure 1(b)). To examine whether treatment of cetuximab combined with cisplatin could potentiate its inhibitory effect on cell growth, HCT116 and SW480 cells were treated with 30 *μ*g/mL cetuximab, with a 1/10 concentration of IC_50_, combined with 2 *μ*g/mL cisplatin, with a 1/2 concentration of IC_50_. We found that 2 *μ*g/mL cisplatin significantly enhanced the inhibitory effect of cetuximab on the cell growth (Figure 1(c)) and density (Figure 1(d)) of HCT116 and SW480 cells, compared to that of 30 *μ*g/mL cetuximab treatment alone, with CI values of less than 1 (0.603 in HCT116 cells and 0.610 in SW480 cells). These results indicate that the combination treatment of cetuximab and cisplatin displays a synergistic inhibitory effect on colon cancer cell growth.

### 3.2. Effects of the Combination Treatment of Cetuximab and Cisplatin on Cell Apoptosis

**Figure 2:**
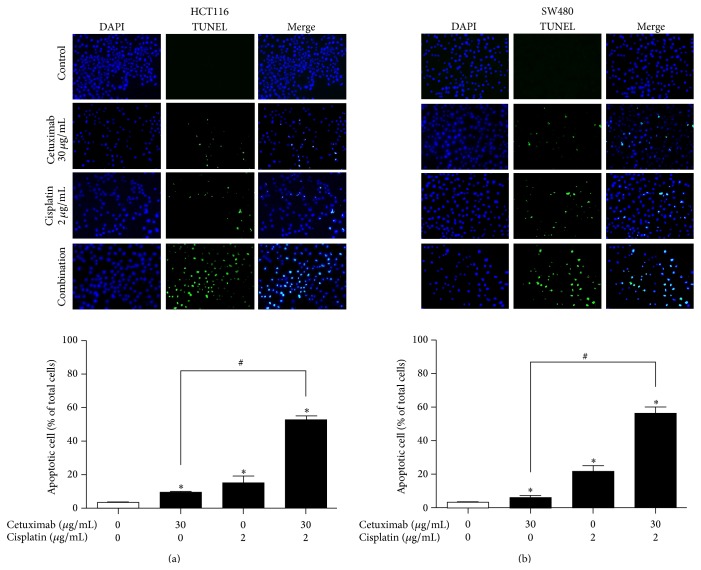
Synergistic effects of cetuximab and cisplatin on apoptotic cell death. (a) HCT116 and (b) SW480 cells were treated with cetuximab (30 *μ*g/mL) or cisplatin (2 *μ*g/mL) alone or a combination of both agents, for 24 h; then, apoptotic cells were examined by TUNEL assay. The total number of cells in a given area was determined by using DAPI nuclear staining (blue color). The green color marks TUNEL-positive cells. The apoptotic index was determined as the TUNEL-positive cell number divided by the total cell number (DAPI-stained cells) under fluorescence microscopy (magnification, 200x). Representative images of each experimental group are shown. The data are expressed as the mean ± SD of three independent experiments. ^*∗*^ *P* < 0.05 indicates statistically significant differences from the control. ^#^ *P* < 0.05 indicates statistically significant differences from the cetuximab treatment alone.

Cell apoptosis contributes to cell growth inhibition [36]; thus, we evaluated the effect of cetuximab combined with cisplatin on apoptotic cell death in HCT116 and SW480 cells using the TUNEL assay. Our results show that treatment of HCT116 (Figure 2(a)) and SW480 (Figure 2(b)) cells with 30 *μ*g/mL cetuximab, which previously showed a mild cell growth inhibition, induced mild cell apoptosis, with values of 9.1% and 6.3%, respectively. Interestingly, we found that treatment of cetuximab combined with cisplatin significantly increased apoptotic cell population in both HCT116 (52.1%) and SW480 (56.4%) cells, compared with a treatment of cetuximab or cisplatin alone.

### 3.3. Effects of the Combination Treatment of Cetuximab and Cisplatin on the EGFR and MAPK Signaling Pathways

**Figure 3:**
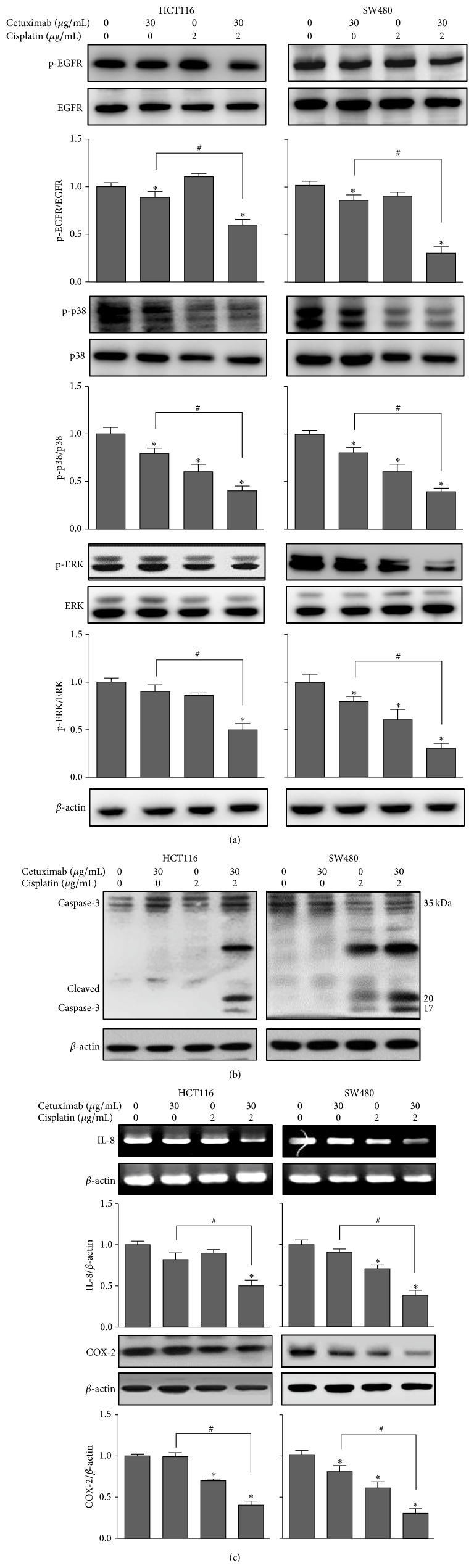
Effects of the combined treatment of cetuximab and cisplatin on the expression of EGFR, MAP kinase, and related signaling proteins. HCT116 and SW480 cells were treated with cetuximab (30 *μ*g/mL) or cisplatin (2 *μ*g/mL) alone or the combination of both agents, for 24 h. (a) Expression of p-EGFR, EGFR, p-p38 MAPK, p38 MAPK, p-ERK, or ERK was detected by Western blotting using specific antibodies. (b) Cleaved caspase-3 was detected by Western blotting. (c) Expression of IL-8 mRNA was detected by RT-PCR using specific primers. Expression of COX-2 was detected by Western blotting. The *β*-actin protein was used as a loading control. After densitometric quantification, data are expressed as the mean ± SD of three independent experiments. ^*∗*^ *P* < 0.05 indicates statistically significant differences from the control. ^#^ *P* < 0.05 indicates statistically significant differences from the cetuximab treatment alone.

The MAPK pathway is a major intracellular pathway activated by EGFR. To characterize EGFR downstream signaling that may correlate with the synergistic inhibitory effects of cetuximab and cisplatin on colon cancer cell growth, we examined whether combination treatment with cetuximab and cisplatin affected EGFR and its downstream signaling pathway. The results in Figure 3(a) show that the treatment of HCT116 and SW480 cells with 30 *μ*g/mL cetuximab or 2 *μ*g/mL cisplatin showed mild or no effect on EGFR phosphorylation. We found that a treatment of cetuximab combined with cisplatin significantly potentiated the inhibitory effect of cetuximab on EGFR phosphorylation compared with a treatment of cetuximab alone. We further found that the treatment of cells with cetuximab combined with cisplatin significantly reduced the expression of p-p38 and p-ERK compared with cells treated with cetuximab alone.

### 3.4. Effects of the Combination Treatment of Cetuximab and Cisplatin on Caspase-3, IL-8, and COX-2

Because a combination treatment of cetuximab and cisplatin in HCT116 and SW480 cells increased the cell apoptotic activity of cetuximab, we examined whether a combination treatment of cetuximab and cisplatin affected the expression of the proapoptotic protein, caspase-3. We clearly demonstrated that cleavage of caspase-3 was dramatically increased by the combination treatment of cetuximab and cisplatin compared with that of cells treated with cetuximab or cisplatin alone (Figure 3(b)). In addition, we found that a combination treatment of cetuximab and cisplatin significantly reduced the expression of IL-8 mRNA and the COX-2 protein in both cells (Figure 3(c)).

### 3.5. Effects of the Combination Treatment of Cetuximab and Cisplatin on AP-1 and NF-*κ*B Activity

**Figure 4:**
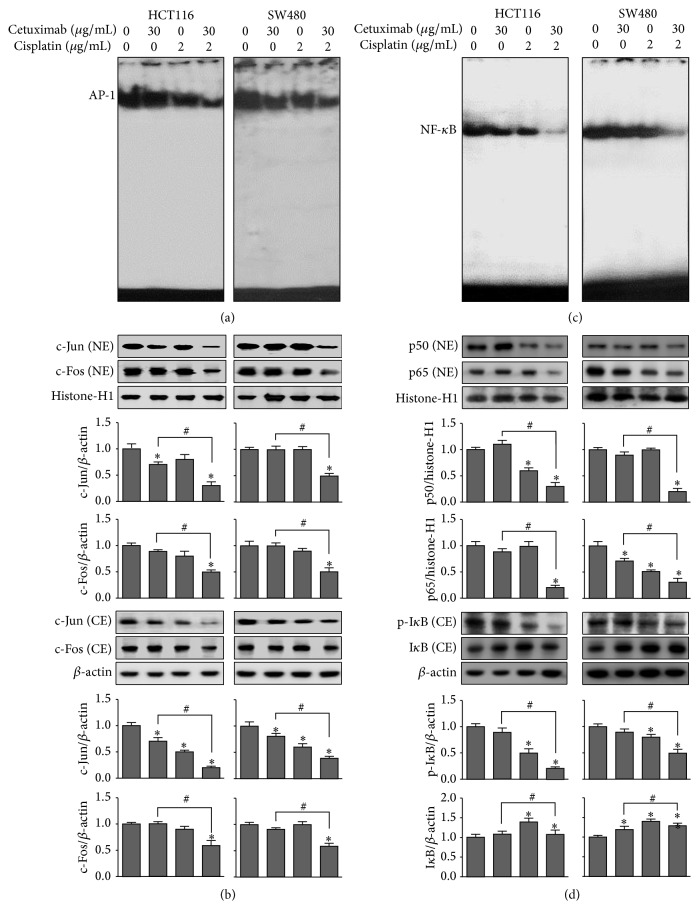
Effects of the combined treatment of cetuximab and cisplatin on AP-1 and NF-*κ*B activity. Cells were treated with cetuximab (30 *μ*g/mL) or cisplatin (2 *μ*g/mL) alone or the combination of both agents. (a) The activation of AP-1 in HCT116 and SW480 cells was investigated by EMSA. (b) Cell protein extracts were fractioned into either nuclear (NE) or cytosolic (CE) extract and the expression of c-Fos and c-Jun in each fraction was detected by Western blotting. (c) The effects of the combined treatment of cetuximab and cisplatin on the activation of NF-*κ*B were investigated by EMSA. (d) The expression of p50, p65, I*κ*B-*α*, and p-I*κ*B-*α* proteins in nuclear (NE) and cytosolic (CE) extracts was detected by Western blotting. The *β*-actin and histone-H1 proteins were used as loading controls. After densitometric quantification, data was expressed as the mean ± SD of three independent experiments. ^*∗*^ *P* < 0.05 indicates statistically significant differences from the control. ^#^ *P* < 0.05 indicates statistically significant differences from the cetuximab treatment alone.

Increased AP-1 and NF-*κ*B activities are implicated in cell survival as well as therapeutic resistance in colon cancer. We thus evaluated the effect of cetuximab combined with cisplatin on AP-1 and NF-*κ*B DNA-binding activity using the EMSA. Our results show that HCT116 and SW480 cells had a strong AP-1 DNA-binding activity, which was strongly attenuated by a combination treatment of cetuximab and cisplatin, compared to that of cells treated with cetuximab or cisplatin alone (Figure 4(a)). In addition, the expressions of c-Jun and c-Fos (components of AP-1) were also significantly inhibited by the combination treatment (Figure 4(b)), which was consistent with the inhibitory effect on AP-1 DNA-binding activity. We also observed a higher level of constitutive activation of NF-*κ*B in both HCT116 and SW480 cells and found that a combination treatment of cetuximab and cisplatin potently inhibited NF-*κ*B DNA-binding activity in both cells (Figure 4(c)). Moreover, we further found that the combination treatment of cetuximab and cisplatin significantly attenuated the nuclear translocations of p50 and p65 through the inhibition of I*κ*B phosphorylation in cell cytosol (Figure 4(d)).

### 3.6. MAPK Pathway Is Involved in the Synergistic Inhibitory Mechanism Underlying the Effect of Cetuximab and Cisplatin on Colon Cancer Cell Growth

**Figure 5:**
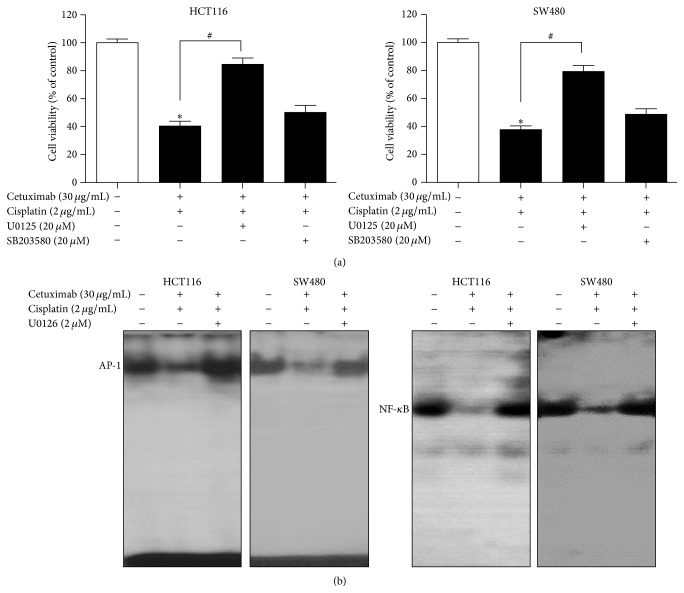
Reversed effect of an ERK inhibitor on the combination treatment-induced cell growth inhibition and downregulation of AP-1 and NF-*κ*B activity. HCT116 and SW480 cells were preincubated with 20 *μ*M of U0126 (an ERK inhibitor) or 20 *μ*M SB203580 (a p38 MAPK inhibitor) for 1 h, followed by incubation with a combination of cetuximab and cisplatin for 24 h. (a) Cell growth changes were determined by MTT assay. (b) HCT116 and SW480 cells were preincubated with 20 *μ*M of U0126 for 1 h, followed by incubation with a combination of cetuximab and cisplatin for 1 h. The activation of AP-1 and NF-*κ*B was determined by EMSA. ^*∗*^ *P* < 0.05 indicates statistically significant differences from the control. ^#^ *P* < 0.05 indicates statistically significant differences from the cetuximab-cisplatin combination treatment.

Because the combination treatment of cetuximab and cisplatin was found to significantly reduce the phosphorylation of p38 and ERK as compared to treatment with cetuximab or cisplatin alone (Figure 3(a)), we further investigated the involvement of the ERK and p38 pathway in the cell viabilities of HCT116 and SW480 cells by employing the ERK and p38 kinase specific inhibitors, U0126 and SB203580, respectively. We found that the pretreatment with U0126, an ERK inhibitor, significantly reversed the synergistic activity of cetuximab and cisplatin on the viabilities of both cells, whereas the pretreatment of SB203580, a p38 inhibitor, caused no statistically significant changes (Figure 5(a)). We further found that AP-1 and NF-*κ*B activities were also reversed by the pretreatment of U0126 (Figure 5(b)), which was consistent with its reversal effect on cell viability. These findings strongly suggest that the ERK pathway might play a critical role in the synergistic inhibitory activity of cetuximab and cisplatin on colon cancer cell growth and viability.

## 4. Discussion

In the present study, the anticancer efficacy of cetuximab combined with cisplatin on cancer cell growth and its action mechanism were evaluated in human colon cancer cells from cell lines HCT116 and SW480. We demonstrated that the combination treatment of cetuximab and cisplatin at a low concentration, which had a mild effect on cell growth and apoptosis, significantly potentiated anticancer activities in both cells. We further demonstrated that the combination treatment with cisplatin significantly enhanced the inhibitory effect of cetuximab on EGFR and MAPK signaling pathway activation, as well as on transcriptional factors and proinflammatory genes. Additionally, we found that the cleavage of caspase-3 was dramatically increased by the combination treatment of cetuximab and cisplatin when compared with that of cells treated with cetuximab or cisplatin alone.

In the past, the US Food and Drug Administration (FDA) approved the use of EGFR-targeted mAbs, cetuximab and panitumumab [37]. These EGFR mAbs are active, mostly in combination with cytotoxic drugs, both in the first line of therapy and in previously treated patients with colon cancer [19]. Cetuximab is used for the treatment of metastatic colorectal cancer and is most often used in combination with irinotecan; however, cetuximab is used alone for patients who cannot use irinotecan or whose cancer is no longer responding to irinotecan [38]. Previous studies demonstrated that cetuximab combined with irinotecan enhanced antitumor activity as compared with cetuximab treatment alone [37, 39]. In the present study, we showed that the combination treatment of cetuximab and cisplatin at a low concentration exhibits a similar synergistic inhibitory effect (1.5-fold increase) on colon cancer cell growth. A previous report demonstrated that the combination of cetuximab and celecoxib significantly reduced EGFR phosphorylation, which contributed to the inhibition of tumor growth in human oral squamous cell carcinoma [40]. Our results also show that a treatment of cetuximab combined with cisplatin effectively suppressed EGFR phosphorylation, while a treatment of cetuximab or cisplatin alone had little to no effect. These results demonstrate that the combination treatment of cetuximab and cisplatin improves anticancer activity by targeting the EGFR pathway in colon cancer cells.

Stimulation of the MAPK pathway, which consists of the ERK and p38 MAPK pathways, by oncogenic proteins or growth factors has been found to be crucial in the development of colon cancer [41]. It is also known that the MAPK pathway is regulated by EGFR signaling in cancer cell growth [42] and that resistance to cisplatin chemotherapy has been shown to involve MAPK signaling [43]. We found that the treatment of cetuximab combined with cisplatin effectively inhibited the ERK and p38 MAPK pathway, which was consistent with its synergistic inhibitory effect on cell growth. Because we observed a correlation between the MAPK pathway and the inhibition of EGFR phosphorylation by the combination treatment of cetuximab and cisplatin in HCT116 and SW480 cells, we then investigated the biological significance of the MAPK pathway in this process. To address this phenomenon, we blocked the activation of the ERK and p38 MAPK pathway by using chemical inhibitors of these pathways. Interestingly, pretreatment with the ERK inhibitor significantly reversed the synergistic activity of cetuximab and cisplatin on the viabilities of both cells, but the same effect was not seen in pretreatment with the p38 inhibitor. Thus, these results suggest that the synergistic effect of cetuximab and cisplatin on human cancer cell growth might be mediated through the inhibition of the ERK signaling pathway rather than* via* the p38 MAPK pathway. We further found that the synergistic activity of cetuximab and cisplatin on the DNA-binding activity of AP-1 and NF-*κ*B was dramatically reversed by pretreatment with the ERK inhibitor. Together, these results demonstrate that the combination treatment of cetuximab and cisplatin suppressed colon cancer cell growth and induced apoptosis by EGFR downregulation* via* the ERK signaling pathway.

IL-8, a chemokine with a defining ELR (glutamic acid-leucine-arginine) amino acid motif, is known to be associated with proliferation, migration, angiogenesis, and chemosensitivity in colon cancer cells and has been shown to be highly expressed in many human tumors, including colon cancer [44]. Many studies have shown that the overexpression and secretion of IL-8 from cells induce the transactivation of EGFR, promoting the downstream activation of MAPK signaling [45, 46]. IL-8 also mediates tumor cell growth and metastasis by binding AP-1 and NF-*κ*B, which have been shown to be associated with several aspects of tumorigenesis and are elevated in colon cancer patients [47, 48]. In the present study, we found that the combination treatment of cells with cetuximab and cisplatin strongly suppressed IL-8 mRNA expression and constitutively activated AP-1 and NF-*κ*B in human colon cancer cells. COX-2 also affects multiple pathways involved in carcinogenesis, including that of colon cancer [49]. Therefore, suppression of COX-2 expression has become an important target for the prevention and treatment of colon cancer [50–52]. EGFR-mediated MAPK signaling pathways are known to play a crucial role in cell proliferation* via* the modulation of COX-2 expression [53]. We found that the expression of COX-2 in colon cancer cells was significantly reduced by the combination treatment of cetuximab and cisplatin. Taken together, our findings demonstrate that the ERK pathway plays a critical role in the synergistic enhancement of the anticancer effects of cetuximab combined with cisplatin on colon cancer cell growth* via* suppression of the expression of p-EGFR along with the inhibition of COX-2, IL-8, and AP-1 and NF-*κ*B.

## 5. Conclusions

In conclusion, the current study shows that the treatment of cetuximab combined with cisplatin in human colon cancer cells exerts synergistic effects on cell growth inhibition and cell apoptosis induction through the inhibition of IL-8 mRNA and COX-2 expression as well as the inhibition of NF-*κ*B and AP-1 activity* via* the attenuation of the ERK-dependent EGFR pathway. Therefore, the combination of cetuximab and cisplatin may be a useful treatment for colon cancer, with a higher effectiveness and fewer reversal effects than other treatments.
